# Itraconazole induced ferroptosis in acute myeloid leukemia cells through TP53INP1

**DOI:** 10.3389/fmolb.2026.1890539

**Published:** 2026-08-26

**Authors:** Yan Qi, Changhao Han, Yizhang Lin, Shidi Cheng, Yuanting She, Meijuan Zhang, Huan Xie, Guangming Yang, Dongfeng Zeng

**Affiliations:** 1 Department of Hematology, Daping Hospital, Third Military Medical University, Chongqing, China; 2 Chongqing Key Laboratory of Hematology and Microenvironment, Chongqing Medical University, Chongqing, China; 3 Department of Weapon Bioeffect Assessment, Research Institute of Surgery, Third Military Medical University, Chongqing, China

**Keywords:** AML, ferroptosis, itraconazole, progression, TP53INP1

## Abstract

**Background:**

Itraconazole (ITRA) is a broad-spectrum antifungal agent approved by the Food and Drug Administration (FDA). It has been shown to inhibit cancer growth, but the underlying molecular mechanism of itraconazole-induced cell death in acute myeloid leukemia (AML) remains largely elusive.

**Methods:**

CCK-8 assay, colony-formation assay, flow cytometry, Western blotting, reactive oxygen species (ROS) detection, and ferroptosis analysis were performed to reveal the role and underlying mechanisms of itraconazole in AML cell lines. Itraconazole has also done research on *in vivo* experiments.

**Results:**

Itraconazole significantly inhibits the viability of AML cell lines and arrests the cell cycle at the G2/M phase. Further investigations reveal that itraconazole effectively induces ferroptosis in AML cells, as evidenced by iron dependency and mitochondrial dysfunction. Notably, the TP53INP1 signaling pathway is markedly upregulated in itraconazole-treated AML cells. Knockdown of TP53INP1 significantly attenuates itraconazole-induced ferroptosis by regulating iron metabolism, which in turn restores mitochondrial function and elevates SLC7A11 and GPX4 levels. Meanwhile, *in vivo* experiments demonstrate that itraconazole impairs AML progression. It is worth noting that concomitant changes in apoptotic markers were also observed, suggesting that itraconazole may simultaneously trigger both apoptotic and ferroptotic pathways, which may coexist in AML cells.

**Conclusion:**

Our findings suggest that itraconazole exerts anti-leukemic effects associated with ferroptosis induction involving TP53INP1, though apoptosis may coexist, supporting its further evaluation as a therapeutic candidate for AML.

## 1 Background

Acute myeloid leukemia (AML) is an aggressive hematological malignancy characterized by the uncontrolled proliferation of immature myeloid cells and impaired differentiation (Zhu et al., 2023). Until recently, treatment options for patients with AML have been limited (Liu, 2021). Although significant therapeutic advances, resistance to initial therapy and disease relapse remain major clinical challenge. Consequently, there is an urgent need to explore and develop more effective and less toxic therapeutic agents.

Itraconazole (ITRA) is a broad-spectrum antifungal agent approved by the U.S. Food and Drug Administration (FDA) (Poirier and Cheymol, 1998) and widely used in clinical practice for fungal infections with well-established safety and efficacy profiles (Boogaerts and Maertens, 2001). In recent years, ITRA has also been found to exert anti-tumor role in various cancers (Chen et al., 2018; Wang et al., 2020; Zhang et al., 2021). Notably, it exhibits concentration-dependent anti-tumor activity in non-small cell lung cancer (NSCLC) by reversing ABCB1-mediated drug resistance (Gerber et al., 2020; Lima et al., 2022), and suppresses esophageal cancer progression through modulation of the HER2/AKT signaling pathway (Zhang et al., 2021). Multiple clinical trials have further demonstrated significant survival benefits in cancer patients (Pantziarka et al., 2015). Despite these promising findings across various cancer, whether ITRA exerts similar efficacy in AML remains unclear.

Ferroptosis is an iron-dependent form of regulated cell death that is mechanistically distinct from apoptosis, necrosis, autophagy, and pyroptosis (Zhang et al., 2022a). This unique cell death modality is characterized by the iron (Fe2+)-dependent lipid peroxidation, depletion of the endogenous antioxidant glutathione (GSH) and altered mitochondria morphology (Liu Y. et al., 2022). In recent years, ferroptosis induction has emerged as a promising anticancer strategy, for instance, bavachin inhibits osteosarcoma cell progression through ferroptosis (Luo et al., 2021) and ZNF498 promotes hepatocellular carcinogenesis by suppressing ferroptosis (Zhang et al., 2022b). Nevertheless, the molecular mechanisms underlying ITRA-induced ferroptosis remain elusive.

Tumor protein 53 induced nuclear protein 1 (TP53INP1), also known as stress induced protein (SIP), is a tumor suppressor gene that is located on chromosome 8q22 (Nishimoto et al., 2019). TP53INP1 is a key regulator of p53 in response to oxidative stress and regulates p73 activity by binding to p53-responsive elements (Tomasini et al., 2005; Saadi et al., 2015). Additionally, TP53INP1 has tumor suppressor activity through regulation of metabolic homeostasis (Saadi et al., 2015). Indeed, TP53INP1 has been reported to act as a tumor suppressor in breast, prostate cancer and colorectal cancer (Giusiano et al., 2012; Liao et al., 2018; Wang et al., 2018). These findings suggest that TP53INP1 functions as a tumor suppressor gene, but its biological function in AML is unknown.

In this study, we demonstrate that ITRA induces ferroptosis in AML cells, at least in part via TP53INP1 upregulation. However, given that apoptotic markers were also observed, we propose that ITRA triggers multiple cell death pathways, with ferroptosis serving as an important contributor to its anti-leukemic activity. These findings support further evaluation of ITRA as a potential therapeutic candidate for AML.

## 2 Materials and methods

### 2.1 AML patient

Primary human AML bone marrow mononuclear cells (BM-MNCs) were isolated from BM samples of AML patients at Daping Hospital using Lymphoprep™ density gradient medium (Thermo Fisher Scientific, Waltham, MA). A total of 9 AML patient samples were included in this study, with a median age of 58 years (range, 37–79) at the time of sample collection, comprising 4 males and 5 females. All samples were obtained from patients with newly diagnosed AML. Cytogenetic risk stratification, evaluated according to the ELN 2022 guidelines, among whom 5 were classified intermediate, and 4 as adverse risk. Common molecular alterations included NPM1 mutations in 2 cases, FLT3-ITD in 3 cases, and IDH1/2 mutations in 2 cases,and NRAS mutations in 2 cases. Of the total samples were used for colony formation assay. All participants were individually informed and consented to participate in the following study. The study was approved by the Ethics Committee of Daping Hospital, Third Military Medical University (Chongqing, China).

### 2.2 Cell culture and transfection

Human AML lines (THP-1, HEL) were purchased from Procell (Wuhan, China), the STR profiling authentication was performed before all experiments and all cells used in this study were routinely tested for *mycoplasma* contamination using a PCR-based detection kit. All results were negative. The cells were grown in RPMI 1640 medium (HyClone) supplemented with 10% fetal bovine serum (HyClone) and 1% Penicillin/Streptomycin (Sigma, St. Louis, MO, USA). Cells were incubated at 37 °C, 5% CO2. Only cells at passages 5–20 after resuscitation were used. All *in vitro* functional experiments were completed within 4 weeks post-revival to avoid phenotypic drift induced by long-term serial passaging.

The TP53INP1 overexpressing plasmid (pcDNA3.1-TP53INP1) and empty vector were obtained from TsingKe (Beijing, China). Cells were planted in 6-well plates at a density of 1*10^5^ cells per well. After 24 h , cells were 70%–80% confluent. The plasmids were then transfected using Lipofectamine® 3,000 (Invitrogen, Carlsbad, CA, USA), in accordance with the manufacturer’s instructions.

### 2.3 Cell proliferation assay

Cell proliferation was assessed using the Cell Counting Kit-8 (CCK-8, MedChemExpress, New Jersey, USA) assay, as described previously (Han et al., 2023; Qi et al., 2023). ITRA treated cells (5,000 cells per well) were seeded into 96-well plates and cultured for 24, 48, 72 and 96 h. After that, 10 μL of CCK-8 solution was added to each well and incubate for 2 hours at 37 °C. Finally, the absorbance at 450 nm was measured using microplate reader (Thermo Fisher Scientific, Waltham, MA).

### 2.4 Cell cycle analysis

THP-1 and HEL cells (1 × 10^6^/well in 6-well plates) were treated with or without ITRA for 48 h, harvested, washed with PBS, and fixed with 75% ice-cold ethanol overnight. After washing, cells were resuspended in 500 μL PBS containing 50 μg/mL RNase A and incubated at 37 *°*C for 30 min. Propidium iodide (PI, 50 μg/mL, BD Biosciences) was added to a final concentration of 5 μg/mL, followed by 15-min incubation in the dark. Flow cytometry was performed on a FACSCantoII (BD Biosciences) collecting ≥ 2 × 10^5^ events per sample. Doublets were excluded by FSC-A/FSC-H and SSC-A/SSC-W gating. Cell cycle distribution was analyzed using FlowJo V10.

### 2.5 Cell apoptosis analysis

THP-1 and HEL cells (1 × 10^6^/well in 6-well plates) were treated with or without ITRA for 48 h, harvested, washed with PBS, and resuspended in 1× Annexin V Binding Buffer at 1 × 10^6^ cells/mL. Cells were stained with Annexin V and 7-AAD (Biolegend) at room for 30 min in the dark, then diluted with Binding Buffer and immediately analyzed on a FACSCantoII (BD Biosciences). A minimum of 2 × 10^5^ events were acquired per sample; doublets were excluded by FSC-A/FSC-H and SSC-A/SSC-W gating. Apoptotic populations were quantified using FlowJo V10.

### 2.6 Colony formation assay

The Colony formation procedure was as previously described (Qi et al., 2023). THP-1 and HEL cells were treated with or without ITRA, cells were plated into 6-well plates using MethoCult H4436 (StemCell Technologies) at a density of 500 cells per well. Fourteen days after plating, the number of colonies was imaged and counted.

### 2.7 Edu (5-ethynyl-2′-deoxyuridine)

The Edu procedure was as previously described (Han et al., 2023). To assess cell proliferation, ITRA treated cells were stained with Edu (BeyoClick™ EdU Cell Proliferation Kit with AlexaFluor 488, Beyotime, Shanghai, China) for 2 h , Subsequently, the cells were fixed with 4% paraformaldehyde for 15 min and permeabilized with 0.3% Triton X-100 permeabilization for another 15 min . The cells added to the Click Reaction Mixture were incubated in the dark room for 1 h and incubated with Hoechst33342 for 10 min . Finally, fluorescence microscopy was used to detect the image of the cells. The percentage of EdU-positive cells was calculated from at least five random fields per well using ImageJ software.

### 2.8 Quantitative real-time PCR (qPCR)

THP-1 and HEL cells (5 × 106/well in 6-well plates) were treated with ITRA for 48 h. Total RNA was extracted using TRIzol reagent (Invitrogen). Reverse transcription was then performed using Takara Bio’s Prime Script RT reagent kit (Tokyo, Japan), and qPCR was performed using SYBR Premix Ex Taq kit (Takara Bio Inc). The 2^−^ΔΔCt method was used for relative quantification. All target genes were normalized to the endogenous reference gene GAPDH. The primer sequences are shown in Supplementary Table S1.

### 2.9 Western blot analysis

The Western blot procedure was as previously described (Qi et al., 2021). Proteins (30 µg per lane) were separated by SDS-PAGE and transferred to PVDF membranes. After blocking with 5% BSA, membranes were incubated with primary antibodies overnight at 4 °C. HRP-conjugated secondary antibodies were used for detection via enhanced chemiluminescence (ECL). The intensity of each target protein was first normalized to its corresponding loading GAPDH. The following anti-human antibodies were used in this study: Bcl-2 (#ab182858; Abcam, Cambridge, United Kingdom), cleaved caspase3 (#ab32042; Abcam, Cambridge, United Kingdom), caspase3 (#ab32351; Abcam, Cambridge, United Kingdom), cleaved caspase9 (#ab2324; Abcam, Cambridge, United Kingdom), caspase9 (#ab18107; Abcam, Cambridge, United Kingdom), SLC7A11 (xCT, #ab175186; Abcam, Cambridge, United Kingdom), SLC3A2 (CD98, #abab307587; Abcam, Cambridge, United Kingdom), GPX4 (#ab125066; Abcam, Cambridge, United Kingdom), and GAPDH (#AF1186; Beyotime, Shanghai, China).

### 2.10 Cell transfection

The si-NC and si-TP53INP1 (TsingKe Biotechnology, Beijing, China) were transfected into HEL or THP-1 cells using Lipofectamine® 3,000 (Invitrogen, Carlsbad, CA, United States) accordance to the manufacturer’s instructions. The target sequence of si-TP53INP1 is shown in Supplementary Table S2.

### 2.11 In vivo studies

The mouse model was established by tail vein injection of 5 × 10^6^ HEL or THP-1 cells into immunodeficient (NOD-SCID) mice (Shanghai Model Organisms Center, China). Approximately 1 week later, the mice were randomly divided into three groups: the DMSO group, ITRA treatment group and ITRA + Fer-1 (ferroptosis inhibitor) group. ITRA and Fer-1 were dissolved in DMSO and then diluted with normal saline (final DMSO concentration ≤ 1%) before use. All groups received intraperitoneal injections three times weekly for 3 weeks, with both drugs administered at a standardized volume of 100 µL per 20 g body weight. At the endpoint, mice were sacrificed. Spleen and liver weights were measured. Tissues were subjected to hematoxylin and eosin (H&E) staining for histopathological analysis. After treatment with ITRA, the survival rates were monitored and Wright-Giemsa analysis of the blast cells in the PB and BM. To further validate the findings, a secondary model was established by injecting the AML cell line C1498 into the tail vein of C57BL/6 mice. All experimental procedures were approved by the Animal Care Committee of the Third Military Medical University (Chongqing, China, Approval no. AMUWEC20247075).

For competitive transplantation assays, 5 × 10^5^ BM cells from C57BL/6 (CD45.2) were mixed with an equivalent number of CD45.1+ BM cells were transplanted into lethally irradiated (10 Gy) CD45.1 mice via intravenous injection. The level of chimerism and the distribution of lineages in the peripheral blood (PB) of the recipient mice were measured 16 weeks after transplantation. All experimental procedures were approved by the Animal Care Committee of the Third Military Medical University (Chongqing, China, Approval no. AMUWEC20247075).

### 2.12 Measurement of intracellular chelate iron

The intracellular chelate iron procedure was as previously described (Gu et al., 2026). THP-1 and HEL cells were seeded at a density of 5 × 10^5^ cells/well in 12-well plates overnight. After treatment with or without ITRA for 48 h , the culture medium was removed, and cells were washed twice with PBS. Subsequently, cells were incubated with 1 μM Phen Green SK diacetate (MedChemExpress) in PBS at 37 °C for 15 min . Following incubation, cells were washed twice with PBS to remove extracellular dye. Then fluorescence microscope was used to examine the image of the cells. For quantification, the mean fluorescence intensity (MFI) was analyzed in at least five random fields per well (≥ 200 cells per field) using ImageJ software, and data are presented as relative fluorescence units normalized to the control group (set as 100%).

### 2.13 Measurement of ROS

THP-1 and HEL cells were seeded at a density of 5 × 10^5^ cells/well in 12-well plates overnight. After treatment with or without ITRA for 48 h , the culture medium was removed, and cells were washed twice with PBS. Subsequently, cells were incubated with serum-free medium containing 10 µM DCFH-DA for 30 min 37 °C and analysed by flow cytometry.

### 2.14 GSH assay

The level of GSH was measured by GSH Assay Kit (Nanjing Jiancheng Bioengineering Institute) according to the instructions.

### 2.15 C^11^-BODIPY assay

THP-1 and HEL cells (5 × 10^5^/well in 12-well plates) were treated with ITRA for 48 h, washed with PBS, and stained with 5 µM C11-BODIPY581/591 (Thermo Fisher) in serum-free medium at 37 °C for 30 min in the dark. Confocal images were acquired 488 nm (oxidized, green) and 561 nm (reduced, red) excitation. The green/red fluorescence ratio was quantified by ImageJ, with background subtraction.

### 2.16 Transmission electron microscopy (TEM)

THP-1 and HEL cells (5 × 10^6^/well in 6-well plates) were treated with ITRA for 48 h, washed with PBS and fixed in 2.5% glutaraldehyde in 0.1 M phosphate buffer (pH 7.4) at 4 °C for 2 h, post-fixed with 1% OsO_4_ at 4 °C for 1 h, dehydrated through graded ethanol, and embedded in Epon 812. Ultrathin sections (60–80 nm) were cut, stained with 2% uranyl acetate and 0.2% lead citrate, and cell sections were photographed using a JEM-1400 (JEOL, Tokyo, Japan) transmission electron microscope.

### 2.17 RNA-seq

Total RNA was extracted from cells in the DMSO control group and the ITRA treatment group, with three biological replicates per group. RNA quality and integrity were assessed using an Agilent Bioanalyzer 2,100, and qualified RNA samples were submitted to Sinotech Genomics Inc. (Shanghai, China) for library construction and sequencing. Libraries were sequenced on the Illumina NovaSeq 6,000 platform using a 150-bp paired-end strategy. Raw reads were trimmed to remove adapter sequences and low-quality bases, and the resulting clean reads were aligned to the GRCh38.102 human reference genome using HISAT2 (v2.2.1) with default parameters, achieving an alignment rate >95% for all samples. Differentially expressed genes (DEGs) were defined as those with an absolute |log_2_ (fold change)| > 1.5 and an FDR-adjusted q-value < 0.05. Gene Set Enrichment Analysis (GSEA) was carried out using GSEA v4.0.3 (Broad Institute, http://www.broadinstitute.org/gsea) and gene sets were obtained from MSigDB (http://www.broadinstitute.org/gsea/msigdb). The raw sequencing data have been deposited in the NCBI Sequence Read Archive (SRA) under the BioProject accession number PRJNA1482602.

### 2.18 Statistical analysis

SPSS 22.0 software (Chicago, IL, USA) and GraphPad Prism 6.0 software (La Jolla, CA, USA) were used for data analysis. Student’s t-test or Wilcoxon rank-sum test was applied for intergroup comparisons between two groups, while one-way ANOVA or Kruskal–Wallis test was adopted for comparisons among multiple groups. Survival differences were analyzed via the log-rank test and Cox regression analysis. All experiments were independently repeated at least three times. All data were presented as mean ± standard deviation (SD). Unless stated otherwise, all statistical tests were two-sided, and a P-value < 0.05 was considered statistically significant.

## 3 Results

### 3.1 Itraconazole inhibits the cell proliferation in AML cells

**FIGURE 1:**
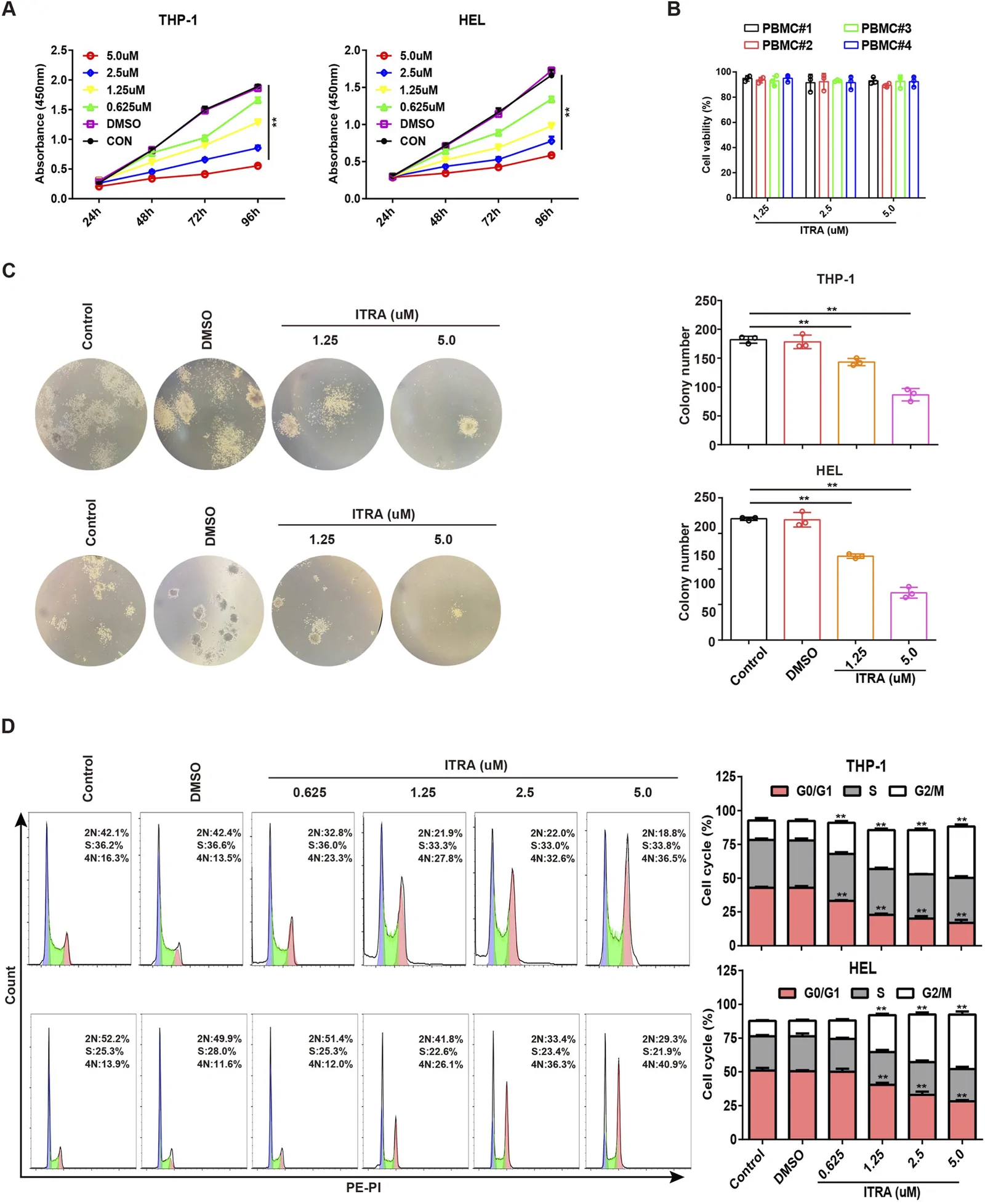
Itraconazole inhibits the cell proliferation in AML cells. CCK-8 assay assessing proliferation of THP-1 and HEL cells treat with ITRA (0.625, 1.25, 2.5, and 5.0 Μm) or control, DMSO for 24, 48, 72, and 96 h **(A)**. CCK-8 assay assessing proliferation of normal peripheral blood mononuclear cell (PBMC) treat with ITRA (1.25, 2.5, and 5.0 Μm) for 48h **(B)**. Colony formation analysis of clonogenic proliferation of THP-1 and HEL cells cultured in control, DMSO, 1.25 μM, and 5.0 μM ITRA for 2 weeks **(C)**. Flow cytometric analysis of the cell cycle in THP-1 and HEL cells cultured in control, DMSO, 0.625 μM, 1.25 μM, 2.5 μM, and 5.0 μM ITRA for 48 h **(D)**. The data are presented as the mean ± SD. All experiments were performed in three independent biological replicates (n = 3). **p < 0.01.

In order to determine the cytotoxic effect of ITRA on the proliferation of AML cell lines, the CCK8 demonstrated that ITRA dose- and time-dependently inhibited AML cell growth, while sparing normal cells (Figures 1A,B). The morphology of the cells under white light was observed (Supplementary Figure S1A). A Dose-dependent suppression of clonogenic proliferation was observed in ITRA treatment AML cells (Figure 1C). Cell cycle analysis revealed that G2/M phases arrest with increasing concentrations of ITRA (Figure 1D) and ITRA caused a significant decrease in EdU-positive cells in the EdU assay (Supplementary Figure S1B). Together, these results are an indication that ITRA acts as a potent inhibitor of AML cell proliferation.

### 3.2 Ferroptosis was triggered by itraconazole in AML cells

**FIGURE 2:**
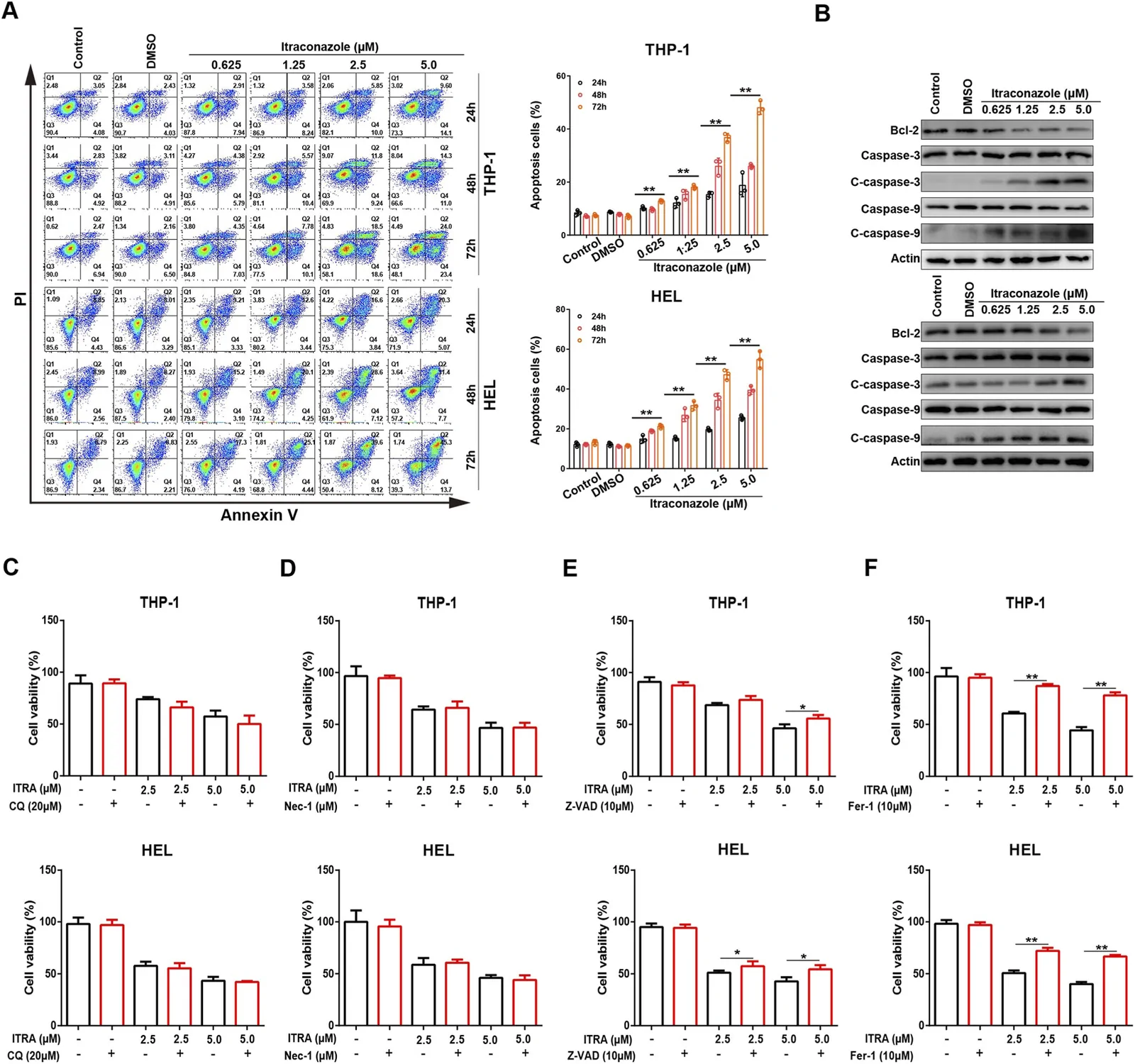
Effect of itraconazole or in combination with various cell death inhibitors on cell viability of AML cells. Flow cytometric analysis of apoptosis frequency in THP-1 and HEL cells cultured in control, DMSO, 0.625 μM, 1.25 μM, 2.5 μM, and 5.0 μM ITRA for 48 h **(A)**. Western blot analysis of Bcl-2, cleaved-caspase3 (c-caspase-3), caspase-3, cleaved-caspase9 (c-caspase9) and caspase9 in THP-1 and HEL cells cultured in control, DMSO, 0.625 μM, 1.25 μM, 2.5 μM, and 5.0 μM ITRA for 48 h **(B)**. CCK8 analysis of cell viability in THP-1 and HEL cells cultured 2.5 μM, and 5.0 μM ITRA with or without CQ (20 μM) for 24 h **(C)**. CCK8 analysis of cell viability in THP-1 and HEL cells cultured 2.5 μM, and 5.0 μM ITRA with or without Nec-1 (10 μM) for 24 h **(D)**. CCK8 analysis of cell viability in THP-1 and HEL cells cultured 2.5 μM, and 5.0 μM ITRA with or without Z-VAD (10 μM) for 24 h **(E)**. CCK8 analysis of cell viability in THP-1 and HEL cells cultured 2.5 μM, and 5.0 μM ITRA with or without Fer-1 (10 μM) for 24 h **(F)**. The data are presented as the mean ± SD. All experiments were performed in three independent biological replicates (n = 3). *p < 0.05, **p < 0.01. Western blot images are representative of three independent experiments.

To systematically investigate the cell death pathways induced by ITRA in AML cells, we initially quantified cell death rates using flow cytometry analysis. As shown that AML cells treated with ITRA led to a significant increase in the percentage of dead cells in dose-dependent manner (Figure 2A). Subsequent Western blot analysis revealed that 24-h exposure to ITRA resulted in dose-dependent upregulation of cleaved caspase-3 (c-caspase-3) and cleaved caspase-9 (c-caspase-9), while reducing the anti-apoptotic protein (Bcl-2) (Figure 2B). To elucidate the specific cell death pathways mediated by ITRA, we employed four pharmacological inhibitors targeting distinct mechanisms: necrostatin-1 (Nec-1, necroptosis inhibitor), chloroquine (CQ, autophagy inhibitor), Z-VAD (pan-caspase inhibitor), and Ferrostatin (Fer-1). However, ITRA-induced cell death was not reversed or slightly reversed by the addition of Nec-1, CQ or Z-VAD alone (Figures 2C–E). Interestingly, Fer-1 significantly rescued cell death induced by ITRA treatment (Figure 2F). Taken together, these results suggest that ferroptosis plays a critical role in ITRA-induced cell death in AML cells, although the concomitant activation of apoptotic pathways indicates that multiple cell death programs may be engaged.

**FIGURE 3:**
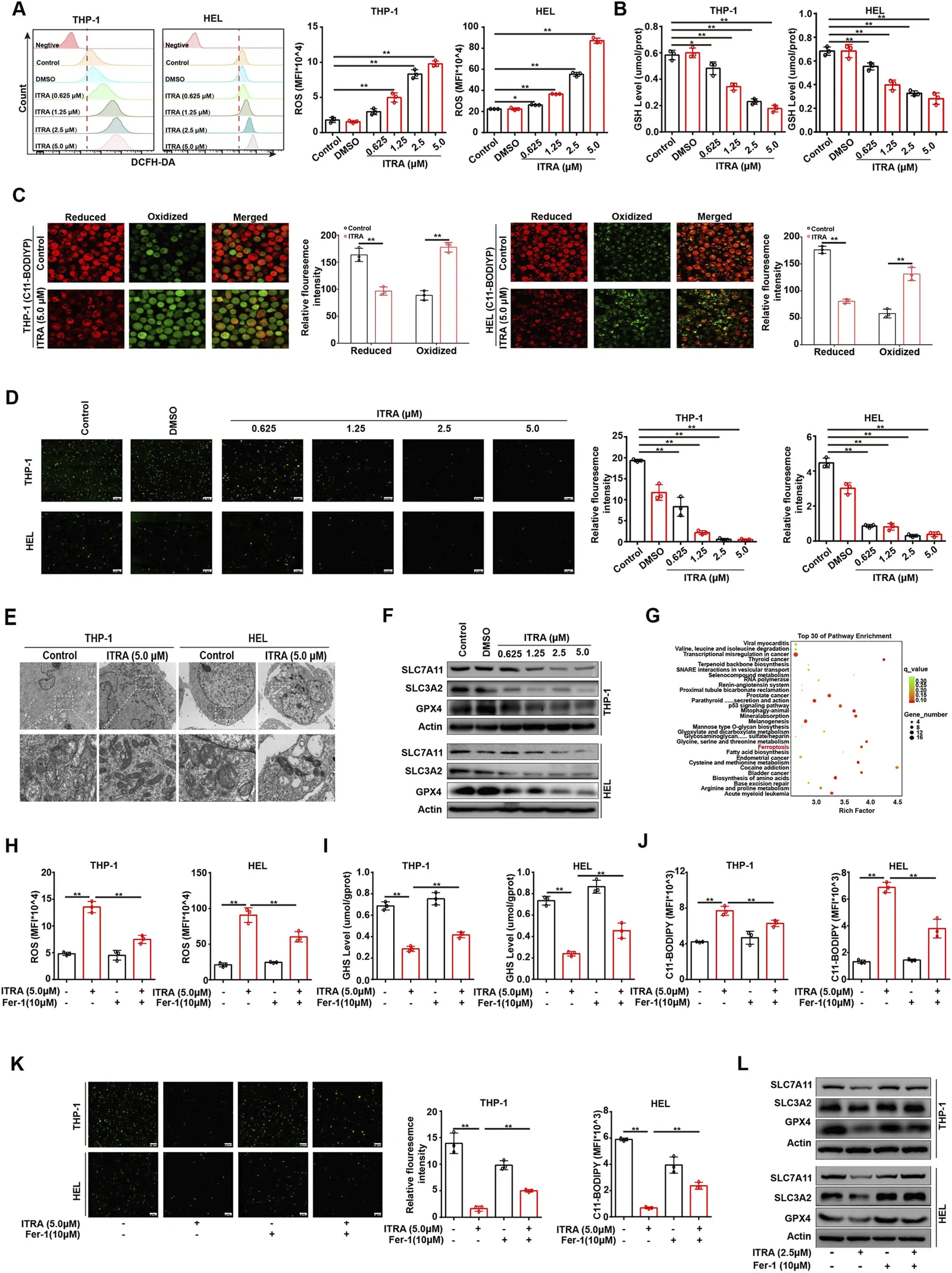
Ferroptosis was triggered by Itraconazole in AML cells. Flow cytometric analysis of ROS levels in THP-1 and HEL cells cultured in control, DMSO, 0.625 μM, 1.25 μM, 2.5 μM, and 5.0 μM ITRA for 48 h **(A)**. GSH Assay analysis of GSH levels in THP-1 and HEL cells cultured in control, DMSO, 0.625 μM, 1.25 μM, 2.5 μM, and 5.0 μM ITRA for 48h **(B)**. Immunofluorescence staining of C11-BODIPY in THP-1 and HEL cells cultured in control, and 5.0 μM ITRA for 48 h **(C)**. Phen Green SK to detect the intracellular chelate iron in THP-1 and HEL cells cultured in control, DMSO, 0.625 μM, 1.25 μM, 2.5 μM, and 5.0 μM ITRA for 48 h **(D)**. Transmission electron microscopy analysis of cell morphology in THP-1 and HEL cells cultured in control, and 5.0 μM ITRA for 48h **(E)**. Western blot analysis of SLC7A11, SLC3A2 and GPX4 in THP-1 and HEL cells cultured in control, DMSO, 0.625 μM, 1.25 μM, 2.5 μM, and 5.0 μM ITRA for 48 h **(F)**. Functional classification of the DEGs selected in the KEGG pathway enrichment analysis through ITRA (5.0 μM) treatment in THP-1 cells for 48 h **(G)**. Flow cytometric analysis of ROS levels in THP-1 and HEL cells cultured 5.0 μM ITRA with or without Fer-1 (10 μM) for 24 h **(H)**. GSH Assay analysis of GSH levels THP-1 and HEL cells cultured 5.0 μM ITRA with or without Fer-1 (10 μM) for 24 h **(I)**. Flow cytometric analysis of C11-BODIPY levels THP-1 and HEL cells cultured 5.0 μM ITRA with or without Fer-1 (10 μM) for 24 h **(J)**. Phen Green SK to detect the intracellular chelate iron THP-1 and HEL cells cultured 5.0 μM ITRA with or without Fer-1 (10 μM) for 24 h **(K)**. Western blot analysis of SLC7A11, SLC3A2 and GPX4 THP-1 and HEL cells cultured 5.0 μM ITRA with or without Fer-1 (10 μM) for 24 h **(L)**. The data are presented as the mean ± SD. All experiments were performed in three independent biological replicates (n = 3). **p < 0.01, Western blot images are representative of three independent experiments.

In order to further verify the involvement of ferroptosis in ITRA-induced cell death, we examined a panel of ferroptosis-specific hallmarks. We found that ITRA treatment significantly increased levels of ROS, C11-BODIPY and decreased levels of GSH (Figures 3A–C; Supplementary Figure S2A). In addition, using the iron-sensitive fluorophore Phen Green SK, we observed decreased proportion of quenched cells following ITRA treatment (Figure 3D), suggesting an increase in intracellular labile iron pools. The microstructure of the cells was then observed using a transmission electron microscope. Smaller mitochondria, higher mitochondrial membrane density and fewer mitochondrial cristae were observed in AML cells treated with ITRA (Figure 3E). Additionally, SLC7A11, SLC3A2 and GPX4 protein levels were downregulated in AML cells after treatment with ITRA (Figure 3F). Consistently, KEGG and GSEA bioinformatics analyses further supported ferroptosis pathway enrichment (Figure 3G; Supplementary Figure S2B). Notably, all these ferroptotic manifestations were effectively reversed by co-treatment with Fer-1 (Figures 3H–L; Supplementary Figure S2C). Taken together, these data indicate that ferroptosis is a major mechanism underlying ITRA-induced cell death in AML, though we cannot exclude the possibility that apoptosis also contributes to this process.

### 3.3 Itraconazole induces ferroptosis by promoting TP53INP1 expression

**FIGURE 4:**
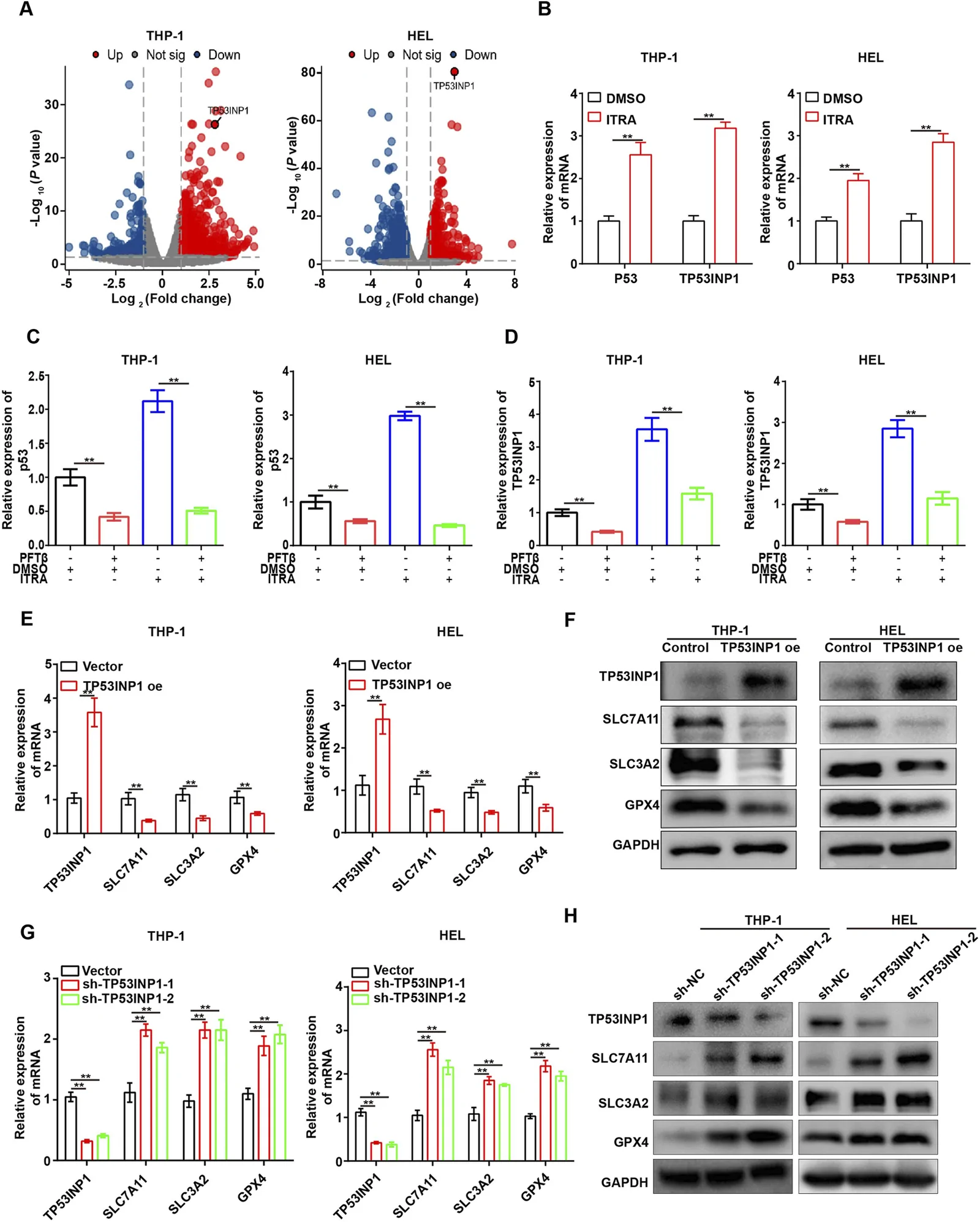
Itraconazole induces ferroptosis by promoting TP53INP1 expression. RNA-seq for identification of differential expression genes (DEGs) between THP-1 cells with and without ITRA. DEGs were identified by fold change >1.5 and FDR p < 0.05 **(A)**. TP53INP1 and p53 levels were analyzed by q-PCR in THP-1 and HEL cells cultured 5.0 μM ITRA for 48 h **(B)**. Q-PCR analysis of the expression of p53 and TP53INP1 in THP-1 and HEL cells after treatment with PFTβ (20 μM) for 48 h **(C,D)**. SLC7A11, SLC3A2 and GPX4 levels in TP53INP1 overexpression THP-1 and HEL cells treated with 5.0 μM ITRA for 48 h were analyzed by q-PCR **(E)** and Western blot **(F)**. SLC7A11, SLC3A2 and GPX4 levels in TP53INP1 knockdown THP-1 and HEL cells treated with 5.0 μM ITRA for 48 h were analyzed by q-PCR **(G)** and Western blot **(H)**. The data are presented as the mean ± SD. All experiments were performed in three independent biological replicates (n = 3). **p < 0.01.

To dissect the molecular mechanisms underlying ITRA-induced ferroptosis in AML cells, RNA-seq analysis was performed on ITRA-treated and untreated AML cells. Interestingly, we have shown that tumor protein p53 inducible nuclear protein 1 (TP53INP1) is significantly upregulated in ITRA-treated AML cells (Figure 4A), a finding confirmed by qPCR in multiple AML cell lines treated with ITRA (Figure 4B). Previous studies have demonstrated that TP53INP1 is a direct target gene of p53 (Alhakamy and Md, 2019; Li et al., 2021). We next examined whether ITRA upregulates TP53INP1 through p53 activation. We observed that the expression of TP53INP1 and p53 were significantly increased in AML with ITRA treatment (Figure 4B). Furthermore, inhibition of p53 using PFTβ significantly abrogated ITRA-induced upregulation of both TP53INP1 and p53 (Figures 4C,D). To further investigate the functional role of TP53INP1 in ITRA-induced ferroptosis, we silenced TP53INP1 expression in AML cells and efficient knockdown was detected by qPCR and Western blot (Figures 4E,F). In addition, we observed that TP53INP1 knockdown significantly restored the mRNA and protein levels of SLC7A11 and GPX4 in AML cells treated with ITRA (Figures 4G,H). Collectively, these findings suggest that ITRA induces ferroptosis in AML cells, at least in part, in a manner involving the p53-TP53INP1 signaling axis, which is associated with downregulating SLC7A11 and GPX4.

**FIGURE 5:**
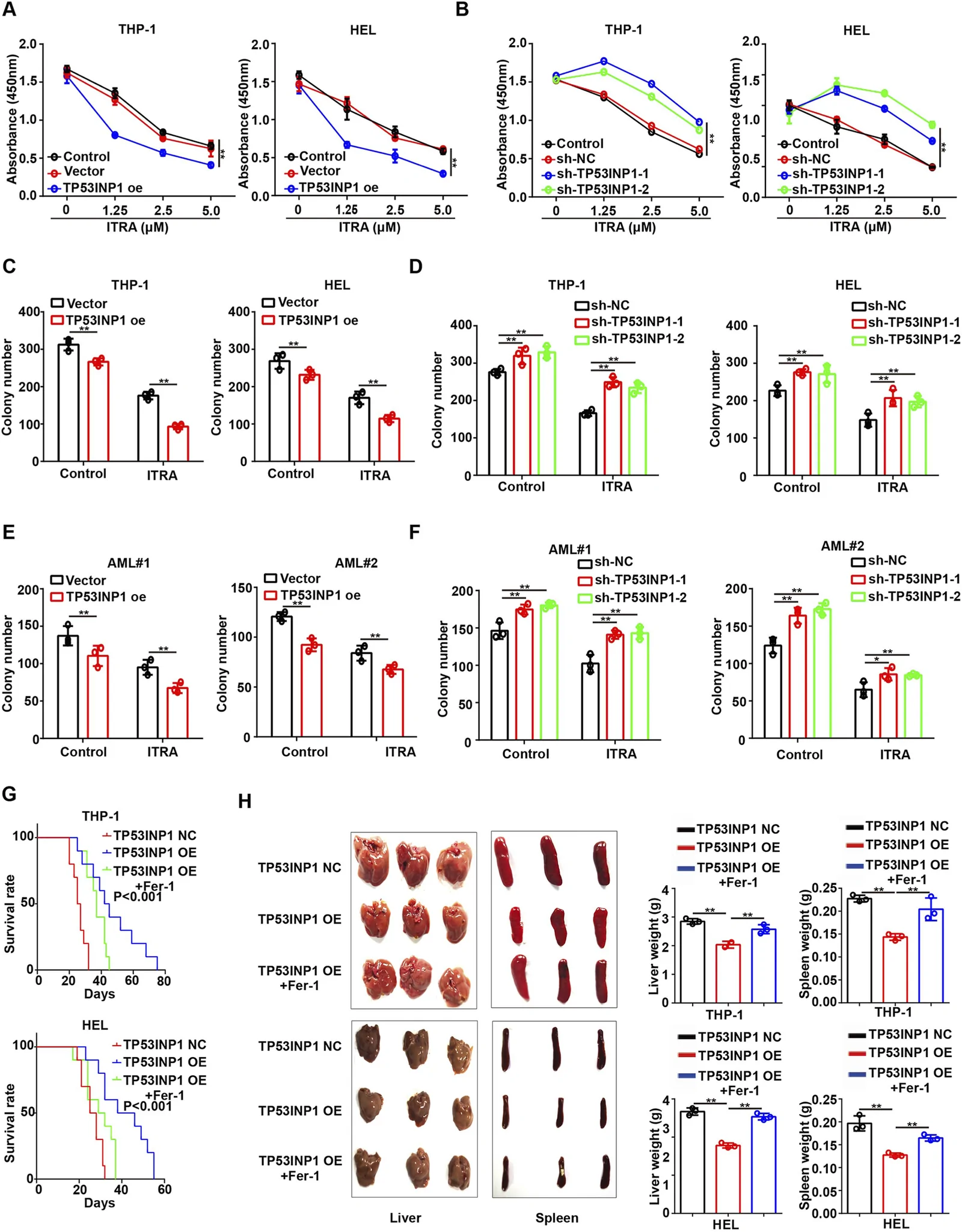
The effect of TP53INP1 on ferroptosis induced by itraconazole in AML cells. CCK-8 assays were performed to evaluate cell proliferation in TP53INP1-overexpressing and TP53INP1-knockdown THP-1 and HEL cells after 48 h treatment with 1.25 μM, 2.5 μM, and 5.0 μM ITRA **(A,B)**. Colony formation analysis of clonogenic proliferation in TP53INP1-overexpressing and TP53INP1-knockdown THP-1 and HEL cells after 2 weeks treatment with 5.0 μM ITRA **(C,D)**. Colony formation analysis of clonogenic proliferation in TP53INP1-overexpressing and TP53INP1-knockdown primary AML cells after 2 weeks treatment with 5.0 μM ITRA **(E,F)**. Kaplan-Meier survival curves were generated to assess the survival duration of NOD-SCID injected with TP53INP1 NC (red), TP53INP1 OE (blue), and Fer-1-treated TP53INP1 OE (Green) THP-1and HEL cells. Log-rank test confirmed significant overall survival differences between groups (n = 10). **(G)** The Liver and Spleen images of mice in different groups 25 days after transplantation. Tissue weight was measured and data are shown on right **(H)**. The data are presented as the mean ± SD. All experiments were performed in three independent biological replicates (n = 3). **p < 0.01.

We subsequently transfected AML cells with TP53INP1 overexpression plasmids or short hairpin RNA (shRNA) to investigate the role of TP53INP1 in itraconazole (ITRA)-induced ferroptosis. CCK-8 and colony formation assays showed that the knockdown of TP53INP1 significantly reversed the anti-proliferative effects of ITRA (Figures 5B,D,F), whereas overexpression of TP53INP1 significantly inhibited the proliferation of AML cells (Figures 5A,C,E). To gain further insight into the *in vivo* role of TP53INP1, we injected NOD/SCID mice via the tail vein with AML cells engineered to have inducible TP53INP1 expression. Our results showed that TP53INP1 overexpression prolonged the survival time of mice (Figure 5G). In addition, the spleen size and liver weight were clearly reduced by the over-expression of TP53INP1 (Figure 5H). However, Fer-1 (a ferroptosis inhibitor) reversed these effects (Figures 5G,H). These results suggest that the tumor-suppressive function of TP53INP1 is mediated, at least in part, through ferroptosis.

Collectively, these findings implicate TP53INP1 as a potential mediator of ITRA-induced ferroptosis in AML, though the concurrent activation of apoptotic pathways (as noted in Figure 2B) suggests that additional cell death mechanisms may also contribute to the overall anti-leukemic effect.

### 3.4 Anti-AML effects of itraconazole in vivo

**FIGURE 6:**
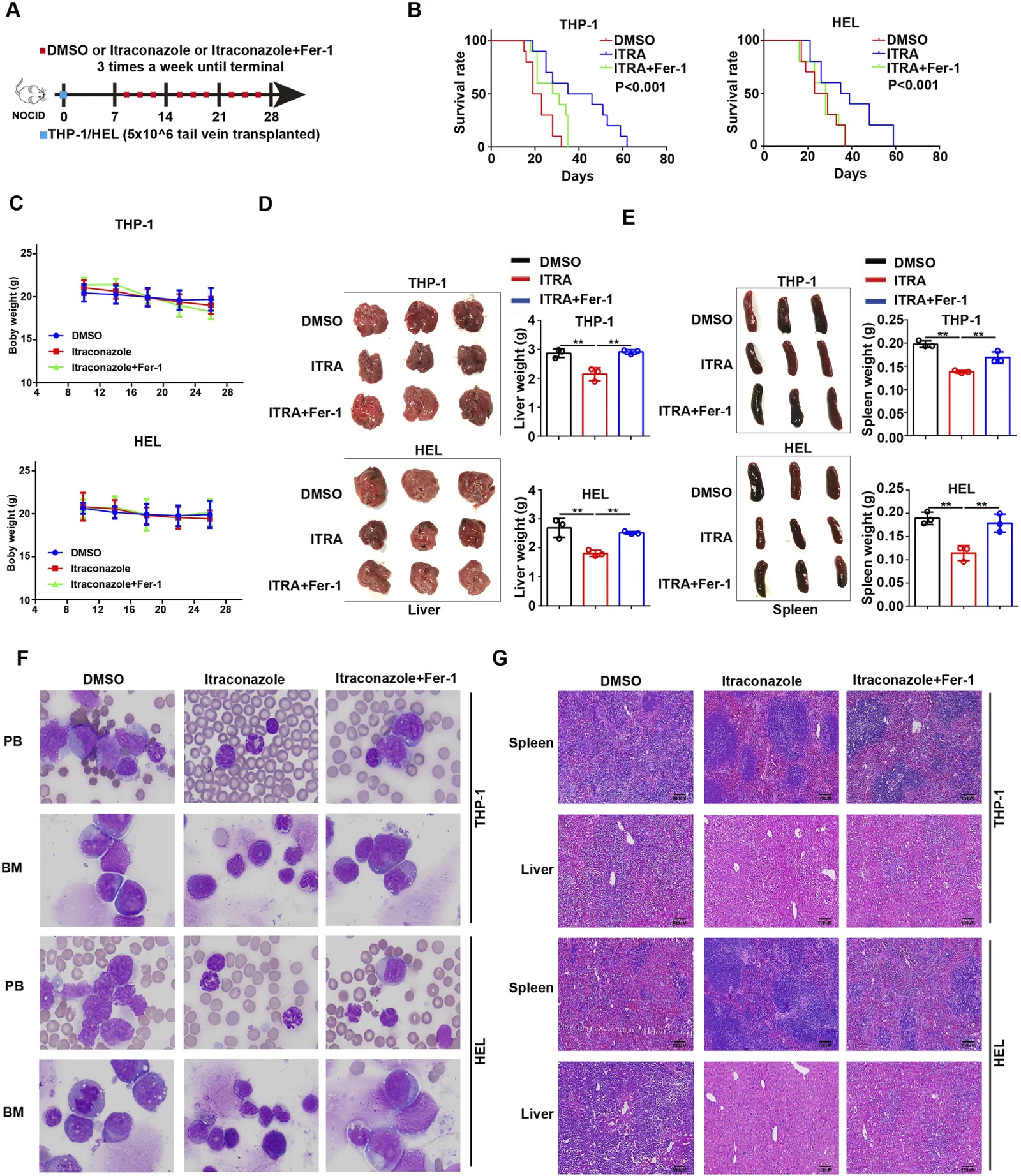
Anti-AML effects of itraconazole *in vivo*. The schematic of NOD/SCID mice transplanted with THP-1 and HEL cells with different treatments (DMSO, 50 mg/kg ITRA, ITRA + 20 mg/kg Fer-1) three times a week until the experiment was terminated **(A)**. Kaplan-Meier survival curves were generated to assess the survival duration of NOD-SCID injected with DMSO (red), ITRA (blue), and ITRA + Fer-1 (Green) THP-1and HEL cells. Log-rank test confirmed significant overall survival differences between groups (n = 10) **(B)**. The average body weights of mice transplanted in DMSO, ITRA, and ITRA + Fer-1 group **(C)**. Liver **(D)** and Spleen **(E)** images of mice in different groups 25 days after transplantation. Tissue weight was measured and data are shown on right. Wright-Giemsa analysis of the blast cells in PB and BM of mice in different groups 25 days after transplantation **(F)**. Typical images are shown. Scale bars, 40 μm. H&E-stained spleens and livers of mice different groups 25 days after transplantation **(G)**. All experiments were performed in three independent biological replicates (n = 3). Scale bar, 100 μm. The data are presented as the mean ± SD of three independent experiments. **p < 0.01.

We then investigated the in vivo efficacy of ITRA in AML. NOD/SCID mice were injected with AML cells via the tail vein. Approximately 1 week after transplantation, ITRA (50 mg/kg) or the same volume of DMSO was injected intraperitoneally three times a week for 3 weeks (Figure 6A). Our results showed that the survival time of mice treated with ITRA was significantly prolonged (Figure 6B), with no significant changes in body weight observed during the treatment period (Figure 6C). It is worth noting that ITRA treated mice exhibited reduce the size and weight of the spleen and liver (Figures 6D,E). Meanwhile, there was a reduction in the number of blast cells in both the PB and BM of mice (Figure 6F), and histopathological analysis further revealed reduced leukemic infiltration of the spleen and liver of ITRA-treated mice (Figure 6G), Importantly, co-administration of fer-1effectively abrogated the aforementioned an-leukemic effects, indicating that ferroptosis contributes to the in vivo activity of ITRA. In addition, we used another murine AML model, C57BL/6J mice transplanted with the murine AML cell line C1498, and consistent results were obtained (Supplementary Figure S3). Collectively, these data indicate that ITRA exhibits potent anti-leukemic activity in vivo, consistent with ferroptosis-dependent mechanisms, although the concurrent apoptotic activation observed in vitro suggests that multiple cell death pathways may collectively contribute to its therapeutic efficacy.

### 3.5 Potential biosafety value of itraconazole in treating tumors

**FIGURE 7:**
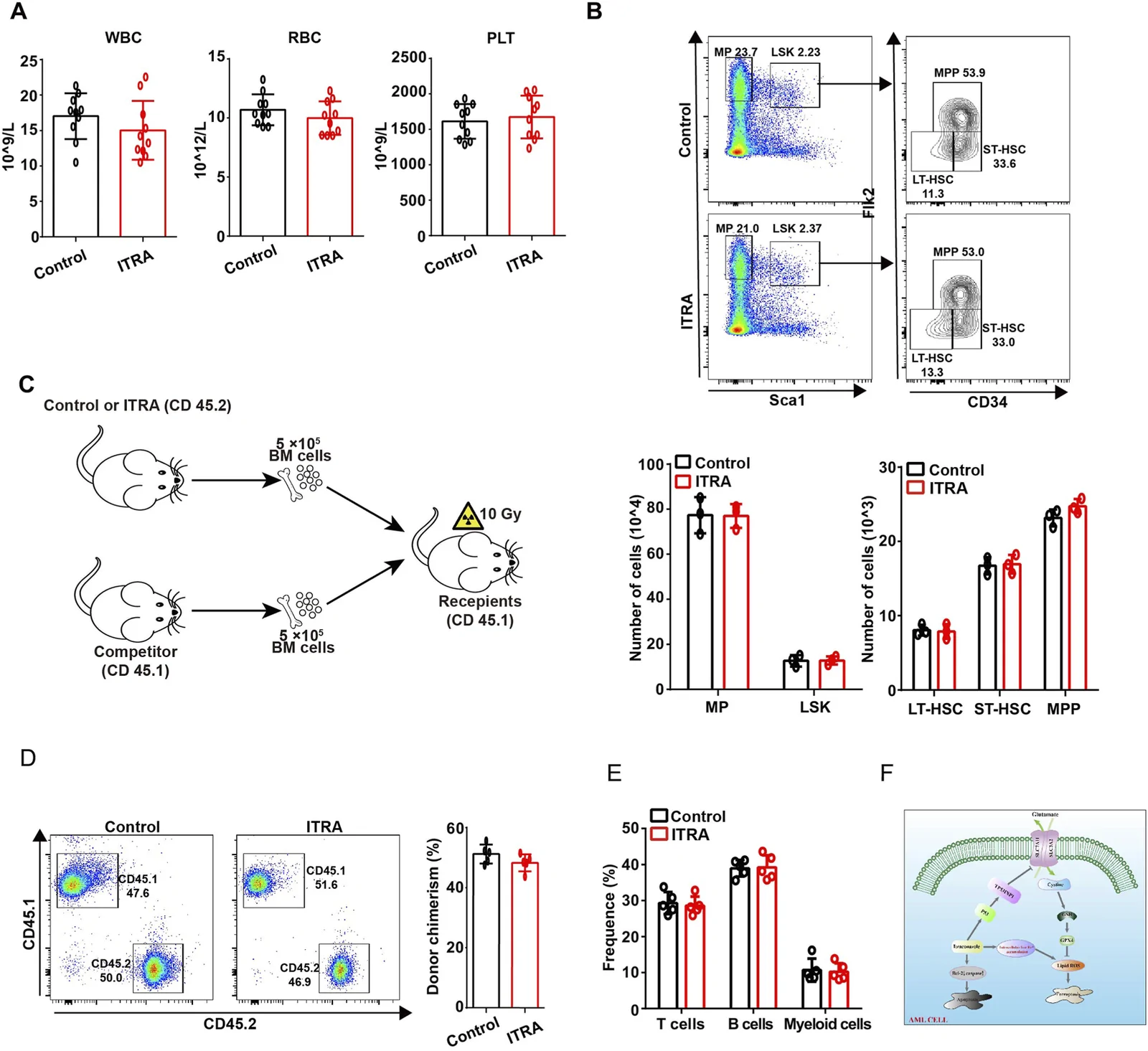
Potential biosafety value of itraconazole in treating tumors. Peripheral WBC, RBC and PLT levels were detected in control and 50 mg/kg ITRA treated mice (n = 10 mice per group) **(A)**. Representative analysis of the percentage and absolute number of MP (Lin− Sca1- c-Kit+), LSKs (Lin− Sca1+ c-Kit+), LT-HSCs (Lin− Sca1+ c-Kit + CD34− Flk2-), ST-HSCs (Lin− Sca1+ c-Kit+CD34^+^ Flk2-) and MPPs (Lin− Sca1+ c-Kit+ CD34^+^ Flk2+) in control and 50 mg/kg ITRA treated mice (n = 3 mice per group) **(B)**. Schematic of competitive transplantation assay **(C)**. Flow cytometric analysis of donor cell (45.2) percentage in peripheral blood of CD45.1 recipient mice 16 weeks post-transplant (n = 5 mice per group) **(D)**. Flow cytometric analysis of T cells , B cells and myeloid cells percentage in donor-derived cells (CD45.2) at 16 weeks after transplantation (n = 5 mice per group) **(E)**. Schematic diagram revealing how ITRA regulates the ferroptosis of AML **(F)**.

Given the established antifungal and emerging anti-tumor properties of ITRA, we next assessed its safety profile with respect to normal hematopoiesis—a critical consideration for any potential therapeutic candidate in AML. The PB of mice treated with or without ITRA was used to measure WBC, RBC and PLT counts, and our results showed that the hematopoietic parameters in two groups did not change (Figure 7A). Additionally, we observed that the percentages and absolute numbers of different subpopulations of Lin-Sca1 + c-Kit + cells (LSKs) and myeloid progenitors (MPs) did not change in ITRA-treated mice compared to untreated mice (Figure 7B). We then carried out a competitive BM transplantation (BMT) in mice treated with or without ITRA (Figure 7C), and found that the percentage of ITRA-treated mice receiving donor-derived cells showed no significant change in the PB of recipients 16 weeks after transplantation (Figures 7D,E). Taken together, these results show that treatment with ITRA does not impair normal hematopoietic function.

Next, hepatic and renal function was measured in sera from mice treated with ITRA and compared with that of the control group. The results showed that the levels of alanine transaminase (ALT) and aspartate transaminase (AST) were not significantly changed by the treatment with ITRA (Supplementary Figures S4A, B). The results of the creatinine (CRE) and blood urea nitrogen (BUN) tests also confirmed the absence of overt kidney dysfunction in the mice treated with ITRA (Supplementary Figures S4C, D). Hematoxylin and eosin (H&E) staining revealed no apparent systemic toxicity in heart, liver, spleen, lung and kidney tissues following ITRA treatment in mice (Supplementary Figure S4E). Altogether, these results indicate that ITRA is well tolerated and has been shown to have some degree of biosafety in mice.

## 4 Discussion

Acute myeloid leukemia (AML) is a clonal neoplasm characterized by the accumulation of leukemic blasts and impaired normal hematopoiesis, and its early clinical manifestations are non-specific and variable (Liu J. et al., 2022). Although new therapies for AML continue to be discovered and applied, many exhibit substantial limitations in antitumor efficacy. (Lier et al., 2022). There is therefore an urgent need for the development of new drugs for AML patients. ITRA has been shown to have anti-tumor activity in several types of cancer in previous studies (Choi et al., 2017; Buczacki et al., 2018; Jiang et al., 2019). However, the precise molecular mechanisms by which ITRA regulates AML progression remain elusive. In the present study, we identify ITRA, a clinically approved antifungal agent, as a potent inducer of ferroptosis in AML cells, and we uncover the p53-TP53INP1-SLC7A11/GPX4 axis as a candidate mechanistic pathway potentially contributing to this effect. These studies suggest that ITRA may be a novel agent for the treatment of AML, with its antitumor activity mediated, at least in part, through TP53INP1-dependent ferroptosis activation ((Figure 7F).

ITRA has been shown to regulate anti-tumor biological processes through various mechanisms (Hu et al., 2017; Deng et al., 2020). ITRA is considered to be an inhibitor of the Hedgehog signaling pathway [25, 27, 28]. The Hedgehog pathway is critical for regulating mitogenic and morphogenic processes during development [29]. Uncontrolled cell proliferation and tumor growth occur when the activities of the Hedgehog signaling pathway become imbalanced [30]. Our study has shown that the Hedgehog signaling pathway has not been altered (Supplementary Figure S5). Studies suggest that ITRA is a promising anticancer agent and has become an area of intense research for the treatment of malignant tumors (Muhldorfer and Konig, 1990; Xu et al., 2021). Study showed itraconazole Inhibits the growth of cutaneous squamous cell carcinoma by targeting HMGCS1/ACSL4 Axis (Xu et al., 2022). This differs from our findings, in which no difference was observed for HMGCS1/ACSL4. We speculate that this regulatory discrepancy may stem from differences in the experimental system; such as ITRA concentration, treatment duration or the cellular microenvironment. Importantly, our mechanistic investigations instead are consistent with the p53-TP53INP1-ferroptosis axis as a pathway potentially involved in ITRA’s anti-leukemic activity, a mechanism not previously linked to ITRA in other malignancies. Although ITRA treatment induced a slight reduction in bone marrow (BM) and peripheral blood (PB) cell counts, both clinical and preclinical evidence supports that this hematological change does not directly increase susceptibility to infection. Researcher have shown that mild to moderate decreases in BM/PB cell counts following ITRA administration are not linked to elevated risk of clinical infections (Horst et al., 1996). Regarding the potential immunosuppressive effects of ITRA, preliminary findings from preclinical models indicate that it does not alter antibody responses or delayed-type hypersensitivity in mice, nor does it cause significant fluctuations in T/B cell counts (Pawelec et al., 1991). Collectively, these findings support the favorable therapeutic window of ITRA and its potential as a novel anti-leukemic agent, with a mechanism of action distinct from that described in other tumor types.

Ferroptosis is a non-apoptotic form of regulated cell death driven by iron-dependent lipid peroxidation, with hallmark features including GPX4 inactivation, SLC7A11 downregulation, and accumulation of reactive oxygen species (Yang et al., 2021). AML cells are highly susceptible to ferroptosis, which constitutes a vulnerability that their survival and progression. To evade ferroptosis upon lipid peroxidation stress, AML cells use multiple adaptive strategies (Zhang et al., 2023). Therefore, there is a need for an understanding of ferroptosis evasion strategies in AML cells. In our study, we observed that ITRA induces ferroptosis in AML cells, as evidenced by increased lipid peroxide accumulation and decreased the protein levels of SLC7A11 and GPX4 and elevated Fe2+ level. Interestingly, RNA sequencing (RNA-seq) analysis revealed that tumor protein p53 inducible nuclear protein 1 (TP53INP1), a stress-responsive apoptotic protein (Giusiano et al., 2012; Liao et al., 2018), was markedly upregulated in AML cells with ITRA treatment. Collectively, these results suggest that ITRA inhibits AML development and may regulate the TP53INP/ferroptosis pathway.

TP53INP1, a stress-responsive gene directly transactivated by p53, may potentiate ferroptosis upstream of this pathway (Li et al., 2024; Wang et al., 2026). P53 centrally controls ferroptosis by repressing SLC7A11 and tuning oxidative stress responses (Wang et al., 2026). We found that TP53INP1 and p53 expression were increased in AML cells treated with ITRA. Additionally, it was observed that p53 inhibitors partially rescued the effects of ITRA on TP53INP1. Furthermore, the knockdown of TP53INP1 markedly decreased ITRA-induced ferroptosis and restored SLC7A11/GPX4 expression, suggesting a model in which TP53INP1 as a downstream effector potentially linking p53 activation to ferroptotic cell death in AML. A particularly noteworthy aspect of our findings is the cellular context in which this regulation occurs. The THP-1 and HEL cell lines used in this study carry mutant TP53 (Bennett et al., 1976; Klemm et al., 2025). In our model, the upregulation of TP53INP1 appears to be primarily driven by ITRA, which may stabilize the mutant p53 protein and facilitate its interaction with the TP53INP1 promoter, thereby circumventing the loss of wild-type p53 function. While the precise mechanism remains to be fully defined, our data suggest that ITRA may exploit a mutant p53-dependent pathway to induce ferroptosis, a finding that could have significant therapeutic implications for AML. Further studies using isogenic wild-type and knockout models are required to fully elucidate the specific contribution of p53 status to this axis.

We acknowledge two key limitations of this study. Our findings rely on AML cell lines without TP53 status characterization or primary sample validation, and the xenograft doses may not be clinically achievable. Mouse models also lack human immune components, limiting translational extrapolation. Mechanistically, while Fer-1 rescue supports ferroptosis, the absence of a full inhibitor panel and genetic rescue leaves apoptotic co-contribution unresolved. Additionally, whether TP53INP1 directly transactivates SLC7A11/GPX4 or acts via p53-dependent/independent programs remains unclear, warranting future ChIP and mutant rescue studies. Despite these limitations, this study provides a mechanistic framework for repurposing itraconazole as a ferroptosis-inducing agent in AML.

## 5 Conclusion

Our findings are consistent with a model in which ITRA induces ferroptosis in AML involving the p53-TP53INP1 axis. However, given the concomitant activation of apoptotic markers and the absence of a comprehensive ferroptosis inhibitor panel, we interpret our data as indicating that ferroptosis is a major, but not exclusive contributor to ITRA-induced cell death. The coexistence of these pathways, along with the potential clinical relevance for p53-mutant AML, warrants further investigation. Nevertheless, the favorable safety profile of ITRA and its efficacy in preclinical models support its further evaluation as a promising therapeutic candidate for AML.

## Data Availability

The raw sequencing data have been deposited in the NCBI Sequence Read Archive (SRA) under the BioProject accession number PRJNA1482602.
