# Supplementary material for: Itraconazole induced ferroptosis in acute myeloid leukemia cells through TP53INP1
Source: Front Mol Biosci. 2026 Aug 26;13:1890539. doi: 10.3389/fmolb.2026.1890539 (PMC13557450; doi:10.3389/fmolb.2026.1890539)
Supplement: Supplementary file 2 [file Supplementaryfile2.docx]

Supplementary materials

Figure S1 (A) The change in cell morphology was observed in THP-1 and HEL cells cultured in control, DMSO, 0.625μM, 1.25μM, 2.5μM, and 5.0μM ITRA for 48h. (B)Immunoﬂuorescence staining of Edu in THP-1 and HEL cells cultured in control, DMSO, 0.625μM, 1.25μM, 2.5μM, and 5.0μM ITRA for 48h.

Figure S2 (A) MDA Assay analysis of MDA levels in HP-1 and HEL cells cultured in control, DMSO, 0.625μM, 1.25μM, 2.5μM, and 5.0μM ITRA for 48h. (B) GSEA revealed enrichment of ferroptosis pathway gene signatures between THP-1 cells with and without 5.0μM ITRA. (C) MDA Assay analysis of MDA levels TP-1 and HEL cells cultured in 5.0μM ITRA with or without Fer-1 (10μM) for 24 h. Data are presented as the mean ± SD of three independent experiments. **p < 0.01.

Figure S3 (A) The schematic of C57BL/6 mice transplanted with AML cells with different treatments (DMSO, 50mg/kg itraconazole, itraconazole+20mg/kg Fer-1) three times a week until the experiment was Terminated. (B) Kaplan-Meier survival curves were generated to assess the survival duration of C57BL/6 injected with DMSO (red), ITRA (blue), and ITRA+Fer-1 (Green) THP-1and HEL cells. Log-rank test confirmed significant overall survival differences between groups (n=10). (C) The average body weights of mice transplanted in DMSO, ITRA, and ITRA+Fer-1 group. Liver (D) and Spleen (E) images of mice in different groups 25 days after transplantation. Tissue weight was measured and data are shown on right. (F) Wright-Giemsa analysis of the blast cells in PB and BM of mice different groups 25 days after transplantation. (G) Typical images are shown. H&E-stained spleens and livers of mice different groups 25 days after transplantation. All experiments were performed in three independent biological replicates (n = 3). Scale bar, 100 μm. The data are presented as the mean ± SD of three independent experiments. **p < 0.01.

Figure S4 The levels of ALT (A) and AST(B), which are markers of liver function, were detected in mice from DMSO, ITRA, and ITRA+Fer-1 treatment groups. The levels of CRE (C) and BUN (D), which are markers of renal function, were detected in mice from DMSO, ITRA, and ITRA+Fer-1 treatment groups. (E) H&E -stained heart, liver, spleen, lung and kidney of mice after transplantation receiving DMSO, ITRA, and ITRA+Fer-1 treatment groups. The data are presented as the mean ± SD of three independent experiments.

Figure S5 The levels of Hedgehog signaling pathway components are not altered by itraconazole treatment in THP-1 cells. Western blot analysis of SHH, PTCH1, SMO, and GLI1 protein levels in THP-1 cells treated with ITRA for 48 hours. The data are presented as the mean ± SD of three independent experiments.

**Supplementary Table 1. qPCR primers used in this study**

| Genes | Forward | | Reverse | |
| --- | --- | --- | --- | --- |
| *TP53INP1* | | 5’- GTGTGCTCTGCTGAGGACTC -3’ | | 5’- GTTGACTTCATAGATACCTGCCC -3’ |
| *SLC7A11* | | 5’- TCCTGCTTTGGCTCCATGAACG -3’ | | 5’- AGAGGAGTGTGCTTGCGGACAT -3’ |
| *SLC3A2* | | 5’- ACCCCTGTTTTCAGCTACGG -3’ | | 5’- GGTCTTCACTCTGGCCCTTC -3’ |
| *GPX4* | | 5’- ACAAGAACGGCTGCGTGGTGAA -3’ | | 5’- GCCACACACTTGTGGAGCTAGA -3’ |
| *GAPDH* | | 5’-TCAAGAAGGTGGTGAAGCAGG -3’ | | 5’- AGCGTCAAAGGTGGAGGAGTG -3’ |

**Supplementary Table 2. shRNA sequences used in this study**

| Genes | shRNA sequences |  |
| --- | --- | --- |
| *TP53INP1-1* | 5’-GGUGGAUUAACCACUAUCAUGAUAGUGGUUAAUCCACC -3’ | |
| *TP53INP1-2* | 5’- CUUGUUGACUUCAUAGAUAUAUCUAUGAAGUCAACAAG -3’ | |

Full western blot images
